# An EEG-Guided Olfactory Interface: Prototype Design and Person-Specific Emotion-Decoding Validation

**DOI:** 10.3390/s26165237

**Published:** 2026-08-19

**Authors:** Jinge Yang, Suihong Lan

**Affiliations:** 1 Creative Computing Institute, University of the Arts London, London SE5 8UF, UK; j.yang0620252@arts.ac.uk; 2 Xiamen Academy of Arts and Design, Fuzhou University, Xiamen 361021, China

**Keywords:** electroencephalography, emotion recognition, affective computing, olfactory display, human–computer interaction, person-specific calibration, adaptive interfaces, digital mental health

## Abstract

**Highlights:**

**What are the main findings?**

- In 39 adults, six induced emotional states were associated with small but FDR-corrected changes in frontal beta power, the beta/alpha ratio and global field power; device-derived on-line metrics showed larger effects.
- Six-class emotion decoding was above chance within participants (45.1%) and remained modest across participants after normalization (23.1%), indicating strong person specificity.

**What are the implications of the main findings?**

- The present study validates the EEG sensing and decoding path only; odor delivery, end-to-end feedback and regulatory efficacy were not experimentally tested.
- A deployable adaptive interface would require per-user calibration, confidence-aware decisions, multimodal robustness testing and quantitative actuator characterization.

**Abstract:**

Just-in-time adaptive interventions require timely and low-burden state estimation, while olfaction offers a programmable output channel with limited attentional demand. We describe a prototype architecture that links electroencephalography (EEG)-based emotion estimation to a six-channel odorant device and evaluate only the EEG sensing and decoding module. Forty EEG sessions from 39 adults were recorded with a 14-channel Emotiv EPOC X headset (128 Hz) during six standardized emotion-induction conditions. No odor was administered. Band-power, frontal alpha asymmetry (FAA) and global field power (GFP) were analyzed with rank-based repeated-measures tests and explicit multiple-comparison correction. Emotion decoding used subject-aware cross-validation. Frontal beta power, the beta/alpha ratio and GFP differed across conditions after false-discovery-rate correction, although effect sizes were small (Kendall’s *W* = 0.089–0.155). On-line affective metrics showed larger effects (*W* = 0.130–0.365). Six-class accuracy was 45.1% ± 13.2% within participants (*n* = 29; chance 16.7%; *p* < 10^−8^) and 23.1% across participants after per-subject normalization (macro-F1 = 0.23; permutation *p* = 0.005). FAA did not differ. Consumer-headset EEG contained person-specific information about laboratory-induced states, but performance was not sufficient to establish a clinically usable regulator. The results validate neither a complete closed loop nor olfactory efficacy; end-to-end latency, artifact and temporal robustness, chemical characterization and controlled odor-regulation effects require prospective evaluation.

## 1. Introduction

Emotional disorders frequently involve difficulties in emotion regulation and impose substantial academic, occupational and social burdens [1,2,3]. Digital interventions, including mobile cognitive–behavioral and mindfulness programs, can broaden access to support [4,5], but their benefit depends on whether assistance reaches the user at an appropriate moment and with an acceptable interaction burden.

Just-in-time adaptive interventions (JITAIs) attempt to match support to a changing state. Meta-analytic evidence indicates small but consistent benefits, while methodological reviews identify persistent weaknesses in trigger definition, availability windows, passive sensing and objective verification of whether the delivered micro-intervention changed the targeted state [6,7]. Prompt-based interventions also require reading, decision-making and deliberate action at moments when distress may reduce those capacities.

Olfaction offers a complementary output channel. Odor processing is closely coupled to limbic and orbitofrontal systems [8], pleasant odors can alter emotional-picture processing [9], and inhalation-aromatherapy studies report mood- and anxiety-related effects, although protocols and formulations remain heterogeneous [10,11,12,13]. Programmable olfactory displays now permit controlled switching, mixing and synchronization [14], making odor a candidate actuator whose formulation, concentration, timing and duration can be specified rather than treated as uncontrolled ambient exposure.

This paper separates system design from empirical validation. First, we describe a prototype architecture that maps an EEG-derived state estimate to a six-channel atomization device with normalization, smoothing, hysteresis and washout logic. Second, we evaluate the sensing path in 39 adults and quantify within- and cross-participant emotion decoding. Third, we prespecify an olfactory-regulation protocol for a future controlled study. No odor was delivered during the reported EEG experiment; therefore, the present evidence does not validate end-to-end feedback or emotion-regulation efficacy.

## 2. Related Work

### 2.1. Eeg-Based Emotion Recognition

EEG-based emotion recognition has used discrete categories and dimensional valence-arousal targets [15,16]. Common representations include band power, differential entropy and frontal asymmetry [17,18], while field-level/topographic measures [19] and functional-connectivity measures such as coherence or phase-locking value and nonlinear-complexity measures can capture inter-regional coupling and signal irregularity that spectral summaries may miss [15,16]. Architectures have expanded from conventional classifiers to CNNs, graph neural networks and transformers that model temporal and spatial structure [20,21,22,23]. However, performance estimates are highly sensitive to label quality, inter-individual variability and data partitioning. Because the present dataset is modest and each emotion was induced in a single block, we use interpretable features and conventional classifiers as a transparent baseline rather than reporting high-capacity models that would be especially vulnerable to block and participant leakage. Connectivity, differential-entropy and deep-model comparisons are identified as prespecified extensions for a multi-block dataset.

### 2.2. Odor, Emotion and Olfactory Interaction

The olfactory and emotional systems are tightly coupled, enabling odors to influence experience, attention and approach-avoidance with comparatively low cognitive demand [8,9]. Randomized and meta-analytic work suggests that inhaled essential oils may affect anxiety, stress or mood, but oil identity, dose, duration, carrier and delivery method vary substantially [10,11,12,13,24]. Multi-scent displays provide a route to reproducible switching and blending [14]. These findings motivate the proposed actuator, but they do not establish that the candidate blends in this study are effective or chemically equivalent across batches.

## 3. Materials and Methods

### 3.1. Proposed System Architecture and Validation Scope

**Figure 1:**
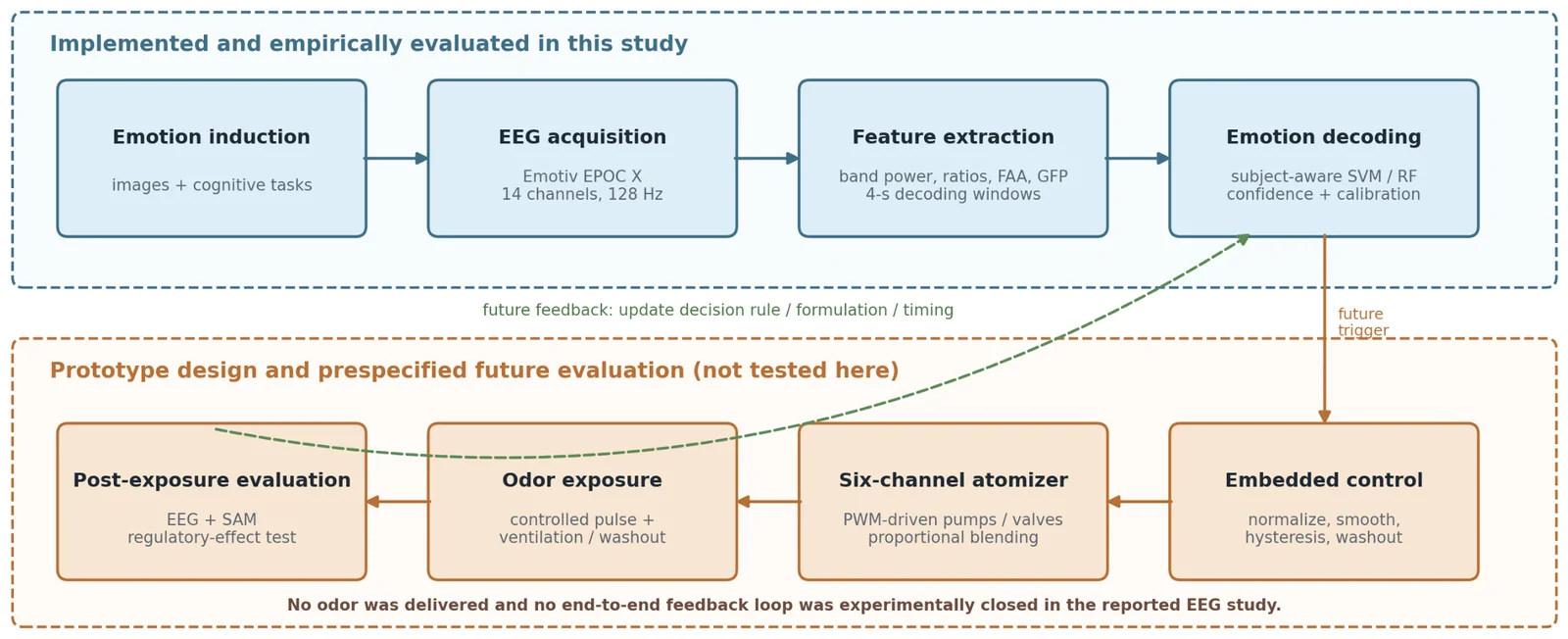
Evidence-scoped architecture of the EEG-guided olfactory interface. The blue upper path was implemented and evaluated in the present study. The orange lower path represents the prototype design and prespecified future protocol. No odor was delivered and no end-to-end feedback loop was experimentally closed in the reported EEG study. The green dashed arrow denotes the proposed future post-exposure feedback path from evaluation back to the decoding/decision stage; this path was not implemented in the present study.

The intended interface contains two evidence levels (Figure 1). The implemented and empirically evaluated path comprises emotion induction, continuous EEG acquisition, feature extraction and emotion decoding. The second path comprises the embedded controller, six-channel atomizer, odor exposure and post-exposure evaluation; this path is represented by a physical prototype and a prespecified protocol but was not tested with participants in the present study. A future end-to-end system would use post-exposure EEG and self-report to update subsequent decisions.

Accordingly, the empirical contribution is restricted to the sensing and decoding path. The atomizer photographs demonstrate prototype construction, not participant exposure or regulatory effectiveness.

### 3.2. Olfactory Output, Embedded Control and Engineering Scope

**Figure 2:**
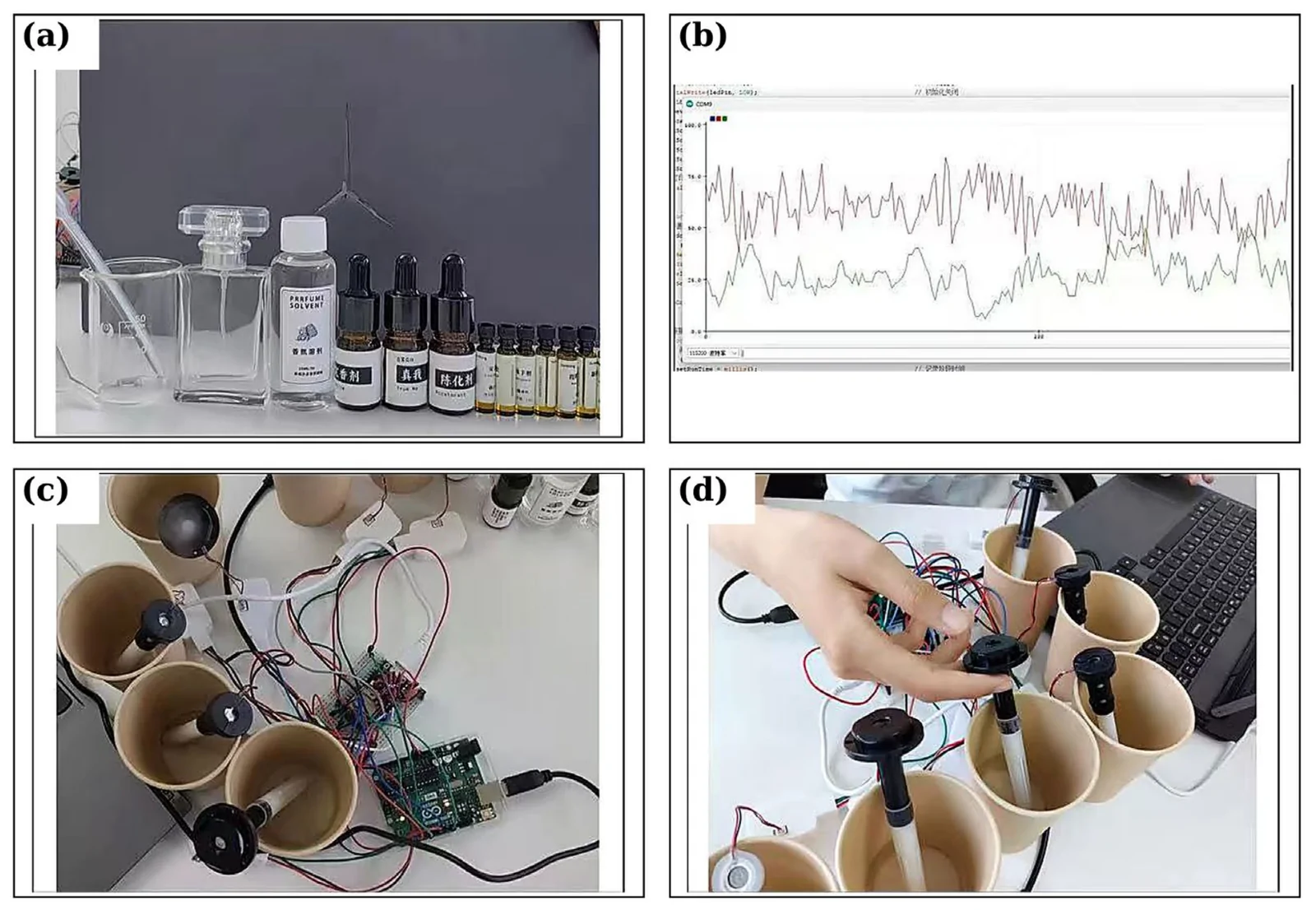
Prototype six-channel odorant apparatus. (**a**) Odorant samples and neutral carrier solvent; (**b**) real-time serial-plotter monitoring during operation, with the red and green lines showing two concurrently displayed monitoring traces (illustrative device signals, not participant EEG outcomes); (**c**,**d**) the microcontroller-driven six-channel atomizer array in delivery cups. Non-English text visible on commercial sample labels in panel (**a**) is incidental and is not required for scientific interpretation.

The prototype contains six independently driven channels (Figure 2). Each channel includes a liquid-supply element, atomizer and airflow path, and an ESP32 microcontroller (Espressif Systems (Shanghai) Co., Ltd., Shanghai, China) modulates channel drive through pulse-width modulation (PWM). Outputs are combined near the outlet. The host-side state estimate is mapped to non-negative channel weights *w*(t), normalized so that Σ*w* = 1, and to a bounded total-intensity term *g*(t). Channel command is therefore u(t) = g(t)w(t). Exponential smoothing and hysteresis are intended to suppress rapid switching, while an explicit ventilation/washout phase accounts for odor persistence. A first-order design model, dC/dt = k u(t) − *C*(t)/*τ*, represents the effective outlet concentration *C*(t), with *τ* summarizing dilution and ventilation.

#### 3.2.1. Synchronization and Quantitative Engineering Characterization

The decoding analysis uses 4 s non-overlapping windows; an implementation based on the same pipeline would therefore have a nominal 4 s state-update interval before computation, communication and actuator delays. For the future trial, the host software is specified to log a common time base for stimulus onset, EEG-window closure, classifier output, controller command and measured atomizer onset. The present study did not measure end-to-end latency, jitter, command loss, channel-to-channel onset dispersion, duty-cycle error, long-duration stability or washout time. We therefore make no real-time reliability claim. These quantities are now prespecified as bench outcomes, following the broader principle that embedded biomedical interfaces should report acquisition, synchronization and reliability metrics rather than architecture alone [25].

#### 3.2.2. Candidate Odor Formulations and Quality-Control Plan

Six candidate essential-oil formulations were defined a priori, one for each target state, using two or three oils at fixed ratios and a planned 5% dilution in a neutral carrier (Table 1). The ratios are protocol inputs derived from prior single-oil and aromatherapy literature [26,27,28,29], not validated therapeutic prescriptions. A neutral carrier serves as the control. The EEG study reported here did not administer any formulation.

No chemical or physical authentication of the oil batches was performed for the present design report. Before efficacy testing, each stock should be documented by botanical name, extraction method, manufacturer, batch identifier and a certificate of analysis. Gas chromatography–mass spectrometry or headspace analysis is the preferred method for volatile composition and delivered-dose characterization; FTIR-ATR combined with multivariate classification can provide rapid identity/adulteration screening, as recently demonstrated for bergamot oil [30]. Infrared thermography with machine-learning analysis may support broader oil-quality screening [31], but it does not replace volatile-compound or headspace-dose measurement. These quality-control steps are now treated as prerequisites for the controlled olfactory study.

### 3.3. Participants

Volunteers were recruited and screened with a pre-study questionnaire assessing baseline affect with the Positive and Negative Affect Schedule (PANAS) and self-reported olfactory function. Inclusion required normal or corrected-to-normal vision, normal olfaction and no neurological disorder or current clinical diagnosis of anxiety or depression. Forty EEG recording sessions from 39 adults were analyzed; one participant was recorded twice. Demographic descriptors were available for 36 questionnaire respondents (18 men and 18 women; mean age 25.8 ± 5.4 years, range 20–43). The difference between the EEG and demographic sample sizes reflects incomplete questionnaire data rather than exclusion from EEG analysis. All participants provided written informed consent, and the protocol was approved by the institutional ethics committee (THU01-20250087).

### 3.4. Eeg Acquisition and Emotion-Induction Paradigm

EEG was recorded with a 14-channel Emotiv EPOC X headset (EMOTIV Inc., San Francisco, CA, USA; AF3, F7, F3, FC5, T7, P7, O1, O2, P8, T8, FC6, F4, F8 and AF4; 10–20 layout; 128 Hz), a consumer system previously validated for event-related-potential research [32]. Sensor-contact and device signal-quality indicators were checked before recording. Each participant completed six counterbalanced induction rounds (anxiety, depression, fear, anger, pleasure and sadness) using affective images and brief cognitive tasks. Each round was flanked by eyes-closed rest; stimuli were presented on a 24-inch monitor (manufacturer and model not recorded) for 6 s within approximately 30 s blocks after a neutral baseline. Self-Assessment Manikin (SAM) ratings were collected descriptively. No olfactory stimulus was delivered. No external EOG, EMG, ECG, electrodermal or respiratory channels were recorded, and the experiment did not include a dedicated noise, movement or longitudinal-drift challenge; these constraints limit artifact and real-world robustness claims.

### 3.5. Feature Extraction and Statistical Analysis

Analyses used device-provided per-channel theta, alpha, low-beta, high-beta and gamma band-power streams together with broadband EEG. For each participant and emotion, epoch averages were used to derive frontal alpha, theta and beta power; occipital alpha; whole-head beta/alpha and theta/beta ratios; FAA = ln(αright) − ln(αleft) for F4/F3 and AF4/AF3; and broadband GFP from 1 to 40 Hz average-referenced EEG. Device-provided engagement, excitement, stress, relaxation, interest, focus and attention streams were summarized separately. Connectivity, differential-entropy and nonlinear-complexity features were not calculated in this analysis and are not implied by the reported results.

The indices are continuous, but their repeated-measures distributions were non-normal; Friedman rank tests were therefore used for omnibus emotion effects, with χ^2^(5), *p* and Kendall’s *W* reported. The Friedman test is not restricted to ordinal outcomes and is appropriate for continuous repeated measures when parametric assumptions are not met. Benjamini–Hochberg correction was applied to the prespecified omnibus outcome panel, and Holm correction was applied only to pairwise Wilcoxon contrasts within outcomes that survived the omnibus correction. No uncorrected pairwise result is interpreted as confirmatory. Topographies use all participants with usable data (*n* = 39); repeated-measures tests use complete six-emotion cases (*n* = 33); on-line metrics use *n* = 27–30 because their proprietary streams update at approximately 0.1 Hz. No a priori power analysis was performed. An approximate sensitivity calculation based on the asymptotic noncentral χ^2^ form of the Friedman statistic indicates that *n* = 33 with six conditions provides 80% power at α = 0.05 for Kendall’s *W* ≈ 0.078; this is reported as a design limitation rather than as retrospective proof of adequacy.

For decoding, each 4 s non-overlapping window contributed 70 features (14 channels × 5 band-power streams). Across retained induction data, 1505 windows were available. A standardized support-vector machine (RBF kernel, C = 10) and a 400-tree random forest were evaluated with (i) participant-grouped five-fold cross-validation for cross-participant generalization and (ii) stratified per-participant cross-validation for within-participant separability. The within-participant analysis included 29 participants with sufficient valid windows in all six classes to construct stratified folds; this explains the difference from *n* = 39 in descriptive topographies. Per-subject z-normalization represents a calibration-assisted setting, not zero-calibration transfer. Accuracy was compared with the 1/6 chance level using 200 label permutations for cross-participant models and a one-sample Wilcoxon test across participants for the within-participant model. Accuracy, macro-F1 and a cross-validated confusion matrix are reported. Analyses used NumPy (https://numpy.org/, accessed on 18 August 2026), SciPy (https://scipy.org/, accessed on 18 August 2026), scikit-learn (https://scikit-learn.org/, accessed on 18 August 2026) and MNE-Python (https://mne.tools/stable/, accessed on 18 August 2026) [33,34,35,36]. Data inclusion and feature construction are summarized in Table 2.

### 3.6. Olfactory-Regulation Evaluation Protocol

The future olfactory-regulation arm is specified as a randomized, blinded within-participant pre/post protocol. After a target state is induced and detected, the matched candidate formulation or neutral carrier would be delivered for 30–60 s while EEG continues; cycles would be separated by ventilation and an empirically determined washout interval. Primary outcomes are pre/post changes in GFP and prespecified band-power indices, complemented by SAM ratings and cluster-based permutation analysis for topographic effects [37]. Before participant testing, the study requires batch-level chemical authentication, headspace-dose characterization, end-to-end latency and reliability benchmarks, and predefined safety and stopping rules. This protocol is reported for transparency; no efficacy result is included in the present paper.

## 4. Results

### 4.1. Induced Emotions Modulate Eeg Band-Power and Field-Level Indices

**Figure 3:**
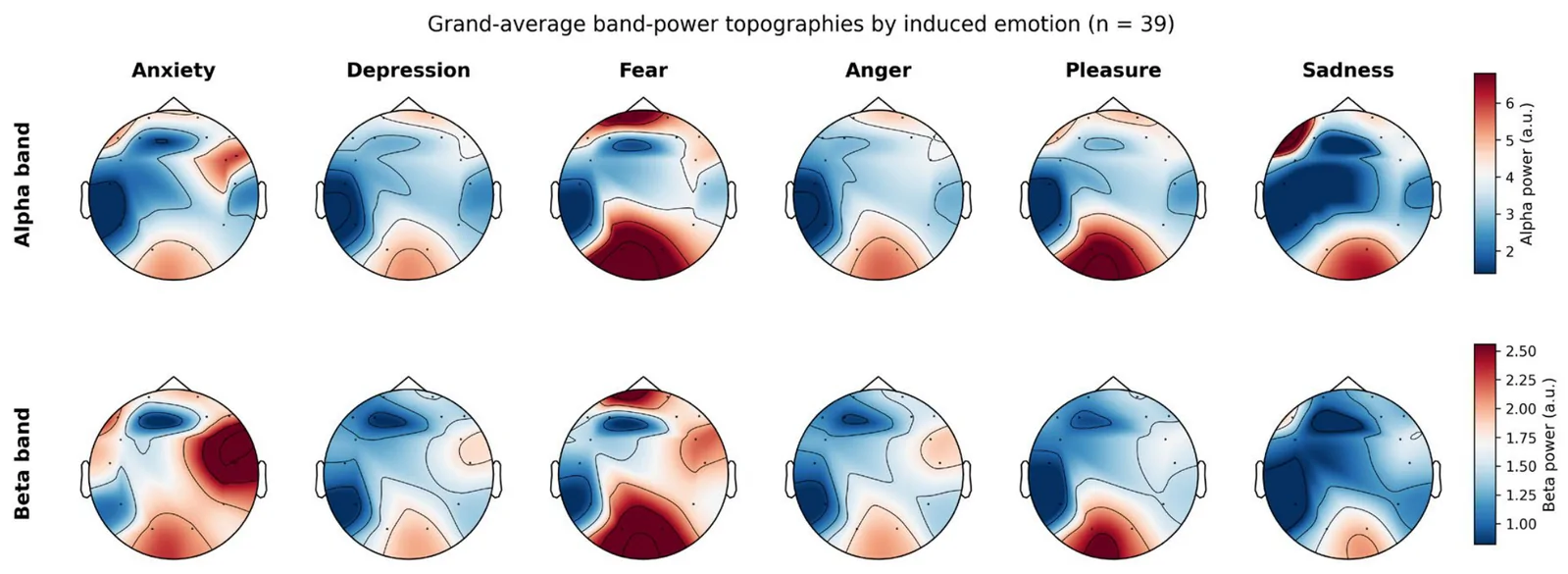
Descriptive grand-average alpha-band (**top**) and beta-band (**bottom**) power topographies for each induced emotion (*n* = 39). The maps visualize spatial patterns and are not, by themselves, inferential evidence of category-specific localization.

**Figure 4:**
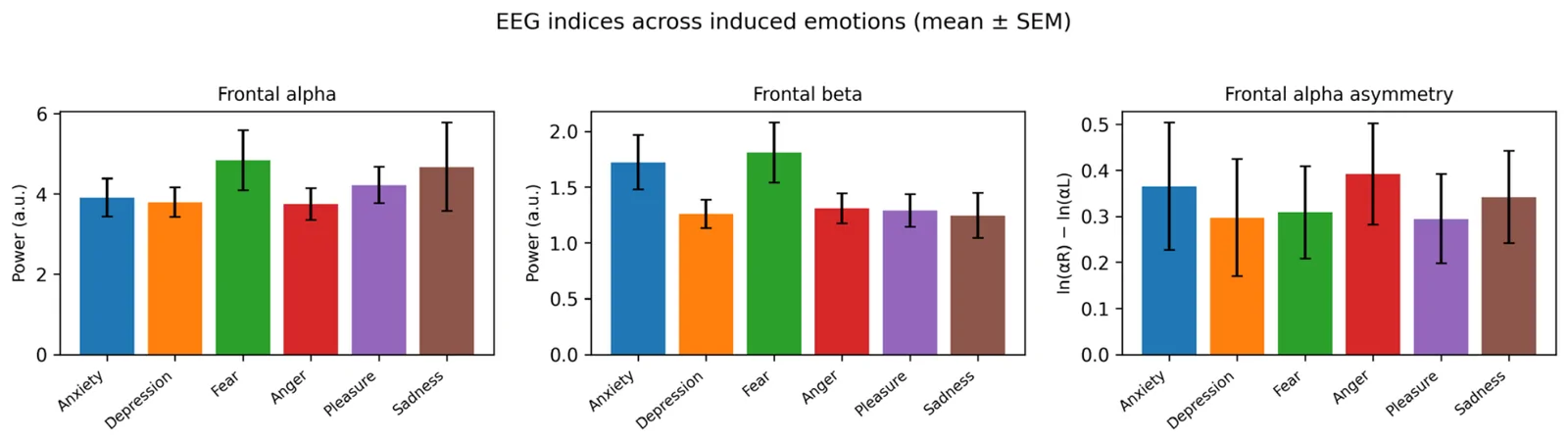
Frontal alpha power, frontal beta power and frontal alpha asymmetry across six induced emotions. Bars show group means and error bars show the standard error of the mean (SEM); SEM is used to display uncertainty around the group mean rather than between-participant dispersion.

Emotion condition was associated with several EEG indices in the 33 complete cases (Table 3). After Benjamini–Hochberg correction, frontal beta power (χ^2^(5) = 14.66, *q* = 0.024, *W* = 0.089), the beta/alpha ratio (χ^2^(5) = 25.64, *q* < 0.001, *W* = 0.155) and GFP (χ^2^(5) = 17.66, *q* = 0.008, *W* = 0.107) remained significant. These *W* values indicate small effects and should not be interpreted as strong emotion-specific biomarkers. Frontal theta and alpha measures did not survive correction. Holm-corrected post hoc contrasts indicated that the beta/alpha ratio was higher for anxiety, depression and anger than for sadness; GFP was higher for fear and pleasure than for sadness and for anxiety than for anger; and frontal beta was higher for fear than for sadness. Table 3 reports omnibus statistics, while Figure 3 and Figure 4 provide descriptive spatial and group summaries.

### 4.2. On-Line Affective Metrics Differentiate Induced States

**Figure 5:**
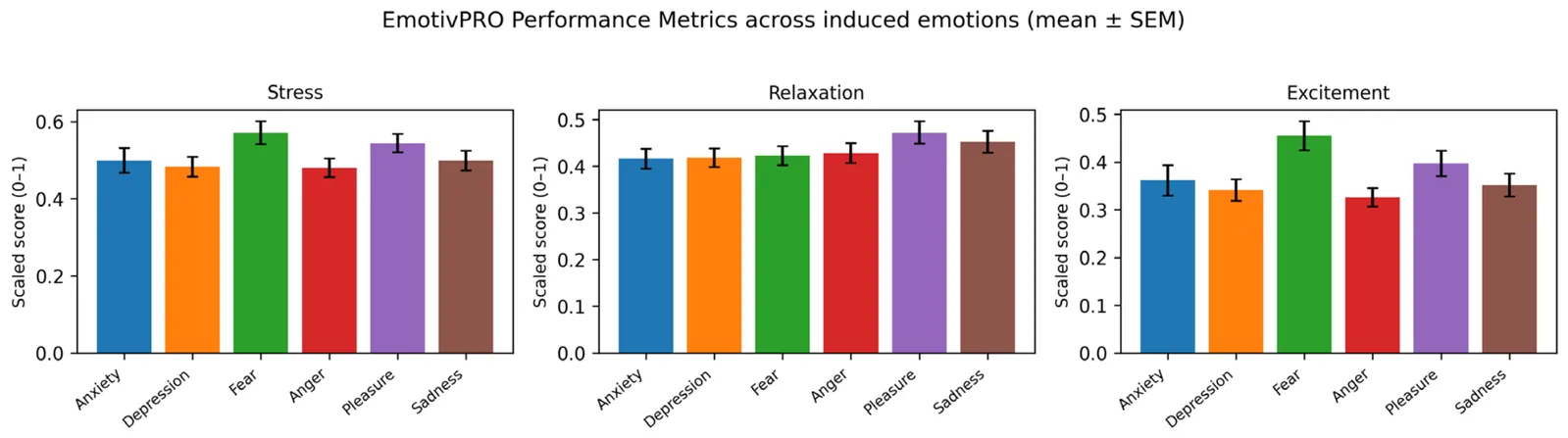
Device-derived on-line affective metrics across the six induced emotions. Bars show group means and error bars show SEM. Relaxation is displayed descriptively; the inferential results are summarized in the text and Table 3.

Five device-derived on-line metrics showed corrected condition effects (Figure 5; Table 3). Engagement had the largest effect (χ^2^(5) = 54.74, *q* < 0.001, *W* = 0.365), followed by interest, excitement, stress and focus (*W* = 0.130–0.266). Holm-corrected comparisons indicated higher excitement for fear than for the other conditions and higher engagement during the more demanding anxiety and fear inductions. These metrics are algorithmically distinct from the manually derived spectral indices but originate from the same EEG stream and proprietary processing; their convergence is therefore supportive, not an independent physiological validation. The pattern may reflect general arousal or task demand as well as discrete emotional state.

### 4.3. Emotion Is Decodable Within—But Not Across—Participants

**Figure 6:**
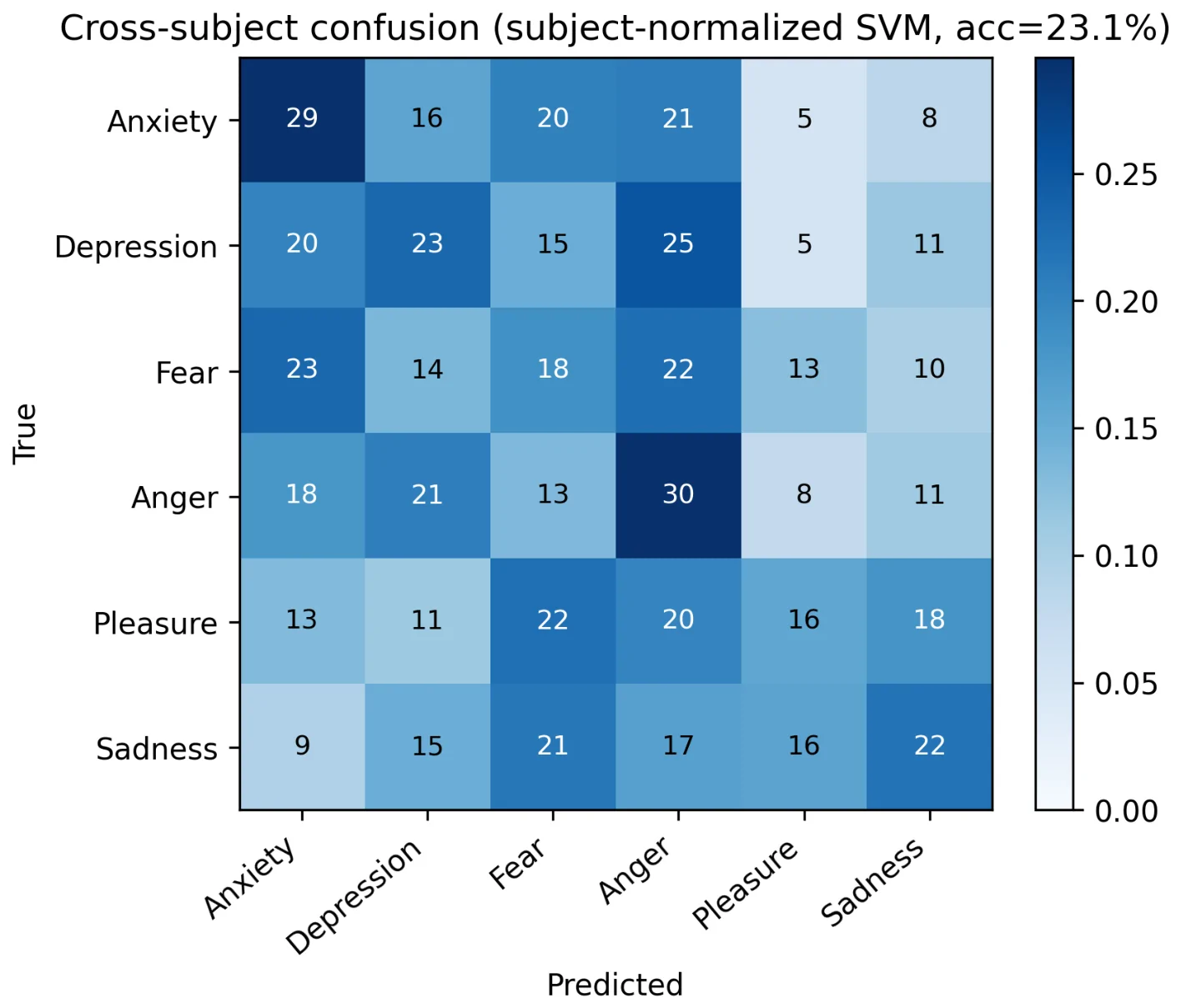
Cross-subject confusion matrix (subject-grouped cross-validation, subject-normalized SVM; overall accuracy 23.1%, chance 16.7%). Cells are row-normalized percentages.

Within-participant six-class decoding reached 45.1% ± 13.2% accuracy in the 29 participants who had enough valid windows in every class for stratified folds (chance 16.7%; Wilcoxon *p* < 10^−8^). Cross-participant performance was 17.7% for the raw-feature SVM and 18.5% for the random forest, increasing to 23.1% after per-subject normalization (macro-F1 = 0.23; permutation *p* = 0.005; Table 4). The normalized result assumes calibration information from the target participant and should not be interpreted as universal zero-calibration transfer. Anxiety and anger were recognized most often, while pleasure was frequently confused with other states (Figure 6). Although above chance, 23.1% cross-participant accuracy and 45.1% within-participant accuracy are insufficient for autonomous clinical decisions. The result supports only a laboratory feasibility claim and motivates personalized calibration, confidence thresholds and an abstention option. Because each emotion was induced in one block, the within-participant estimate remains an upper bound.

### 4.4. Frontal Alpha Asymmetry Did Not Track Induced Emotion

In contrast to the activation indices, frontal alpha asymmetry did not differ across the induced emotions for either electrode pair (F4/F3: χ^2^(5) = 1.62, *p* = 0.898; AF4/AF3: χ^2^(5) = 2.26, *p* = 0.812), and the pre-specified valence contrast (pleasure vs anxiety) was non-significant (Wilcoxon *p* = 0.65). This null result is consistent with reports that FAA is a noisy, trait-loaded index whose state sensitivity is modest, and it argues against relying on FAA alone as the control variable for a real-time system.

## 5. Discussion

### 5.1. Principal Findings

We designed an EEG-guided olfactory-interface prototype and empirically evaluated its EEG sensing and decoding path. Condition effects were detectable in frontal beta power, the beta/alpha ratio, GFP and several device-derived metrics, but the spectral effects were small. Decoding was substantially better within than across participants, while FAA was not state-sensitive. These findings identify person specificity and calibration as central engineering constraints; they do not demonstrate end-to-end feedback, odor-mediated regulation or clinical usability.

### 5.2. Interpretation, Alternative Explanations and Design Implications

The within- versus cross-participant gap suggests that a single population decoder is unlikely to be sufficient. A practical system would require a brief calibration phase, participant-specific normalization or transfer learning, and confidence-aware decisions. Given the modest accuracies, the controller should be permitted to abstain rather than trigger odor when confidence is low, and no high-stakes clinical action should depend on the EEG classifier alone. Multimodal physiological sensing can improve robustness by combining central and peripheral information; recent EEG-EMG adaptive-system work illustrates the value of synchronized fusion and explicit latency reporting [38], although emotion-regulation performance must be evaluated directly in the target context.

Several alternative interpretations remain. The six conditions differed not only in intended emotion but also in visual content, arousal and cognitive demand, so beta and engagement effects may reflect nonspecific task activation. The device-derived metrics are computed from the same EEG stream and are not independent validation. Single-block induction also confounds emotion with time and block identity, and per-subject normalization uses target-participant distributional information. A multi-block, repeated-session design with held-out days is needed to separate emotional state from block, session and task effects.

### 5.3. Olfaction as a Candidate Parameterized Actuator

A programmable atomizer can represent odor as a reproducible control input defined by channel ratio, intensity, pulse duration and washout, rather than as uncontrolled ambient exposure. That engineering capability is distinct from therapeutic efficacy. The candidate blends in Table 1 were not administered, their batch chemistry was not characterized and their emotion-specific effects are unknown. The next study must therefore combine blinded neutral-carrier control, chemical and headspace characterization, measured timing and washout, and objective pre/post outcomes before any regulatory claim is made.

### 5.4. Limitations

Several limitations define the evidentiary boundary. First, only the sensing and decoding path was tested; no odor was delivered, the feedback loop was not closed and regulatory efficacy is unknown. Second, the prototype lacks measured end-to-end latency, jitter, control stability, reliability and washout parameters. Third, the 14-channel consumer headset has limited spatial resolution, proprietary derived streams and no auxiliary ocular or muscle channels; robustness to movement, EMG contamination, packet loss and longitudinal drift was not quantified. Fourth, no a priori power analysis was performed, although a post hoc design-sensitivity estimate is reported. Fifth, the feature set was intentionally simple; functional connectivity, differential entropy, nonlinear dynamics and multimodal physiology were not evaluated. Sixth, one block per emotion can inflate within-participant decoding through time and block confounding. Seventh, young healthy adults and laboratory inductions limit clinical and ecological generalization. Finally, the oil formulations were literature-informed candidates without chemical authentication or delivered-dose measurement.

### 5.5. Applications and Outlook

Future work should proceed in stages: (i) multi-block and repeated-session EEG collection; (ii) artifact and temporal-robustness stress tests; (iii) comparison of spectral baselines with coherence/phase-locking, differential entropy and nonlinear features; (iv) subject-grouped evaluation of CNN, graph and transformer models; (v) multimodal fusion with ECG/heart-rate variability, electrodermal activity, respiration or EMG; (vi) quantitative bench characterization of latency, synchronization, stability and washout; and (vii) a preregistered, blinded odor-versus-carrier trial using chemically authenticated formulations. Only after these stages should clinical, rehabilitation or digital-mental-health applications be considered.

## 6. Conclusions

We presented an EEG-guided olfactory-interface prototype and validated only its EEG sensing and emotion-decoding module in 39 adults. Laboratory-induced conditions produced small corrected changes in frontal beta power, the beta/alpha ratio and GFP, and emotion decoding was person-specific: 45.1% within participants and 23.1% across participants after normalization. The study therefore provides a cautious engineering baseline for personalized state estimation, not validation of a complete closed loop, olfactory regulation or clinical deployment. End-to-end hardware characterization, robust multimodal sensing, chemically controlled odor delivery and a blinded efficacy trial remain necessary.

## Figures and Tables

**Table 1:** Compound essential-oil formulations designed for each target emotion.

Target Emotion	Oil Combination	Design Rationale	Ratio
Anxiety	Lavender + Sweet orange + Patchouli	Selected from reports of calming, anxiolytic or autonomic-modulating effects; the blend remains an unvalidated candidate formulation.	50/30/20
Depression	Bergamot + Jasmine + Rose	Selected from reports of mood-related and pleasantness effects; no neurotransmitter-specific mechanism is assumed in this study.	40/40/20
Fear	Vetiver + Cedarwood + Agarwood	Selected to provide a low-arousal woody profile intended for grounding; regulatory efficacy has not yet been tested.	40/40/20
Anger	Chamomile + Sandalwood + Bergamot	Selected from reports of calming and arousal-reducing effects; the ratio is a protocol parameter rather than an established therapeutic dose.	50/30/20
Pleasure	Lemongrass + Peppermint + Neroli	Selected to provide an invigorating, high-clarity profile while retaining a pleasant citrus/floral component.	40/30/30
Sadness	Jasmine + Frankincense + Thyme	Selected from reports of pleasantness and mood-related effects; the blend requires controlled efficacy and safety testing.	50/30/20

**Table 2:** Data inclusion and feature construction across analyses.

Analysis Component	Data Retained	Construction or Inclusion Rule
Recorded dataset	39 adults; 40 sessions	One adult completed a second recording session.
Descriptive topographies	*n* = 39	All participants with usable emotion-epoch band-power data.
Omnibus repeated-measures tests	*n* = 33	Complete valid data for all six emotion conditions.
On-line affective metrics	*n* = 27–30	Metric-specific availability at approximately 0.1 Hz update rate.
Decoding windows	1505 windows	Non-overlapping 4 s windows from retained induction data.
Features per window	70	14 EEG channels × 5 band-power streams.
Within-participant decoding	*n* = 29	Sufficient valid windows in every class for stratified folds.
Cross-participant decoding	1505 windows	Five folds grouped by participant; no participant appears in both train and test sets.

**Table 3:** Omnibus emotion effects (Friedman tests) on EEG indices and on-line affective metrics. *q* = Benjamini–Hochberg false-discovery rate; bold *q* < 0.05. Effect sizes are Kendall’s *W*.

Index	χ^2^(5)	*p*	*q* (BH)	Kendall’s *W*	*n*
Frontal beta power	14.66	0.012	0.024	0.089	33
Beta/alpha ratio	25.64	<0.001	<0.001	0.155	33
Global field power (GFP)	17.66	0.003	0.008	0.107	33
Frontal theta power	12.34	0.030	0.054	0.075	33
Frontal alpha power	10.45	0.063	0.101	0.063	33
Occipital alpha power	9.48	0.091	0.133	0.057	33
Frontal alpha asymmetry (F4/F3)	1.62	0.898	0.898	0.010	33
Engagement (on-line)	54.74	<0.001	<0.001	0.365	30
Interest (on-line)	38.52	<0.001	<0.001	0.266	29
Excitement (on-line)	31.39	<0.001	<0.001	0.209	30
Stress (on-line)	25.75	<0.001	<0.001	0.178	29
Focus (on-line)	18.91	0.002	0.005	0.130	29

**Table 4:** Six-class EEG emotion-decoding accuracy under different cross-validation schemes (chance = 16.7%). Significance versus chance used a label-permutation test for cross-participant models and a one-sample Wilcoxon test across participants for the within-participant model.

Evaluation Scheme	Model	Features	Accuracy	Macro-F1	*p* vs. Chance
Cross-subject (grouped CV)	SVM (RBF)	Raw band power	17.7%	0.17	*n*.s.
Cross-subject (grouped CV)	Random forest	Raw band power	18.5%	0.18	*n*.s.
Cross-subject (grouped CV)	SVM (RBF)	Per-subject normalized	23.1%	0.23	0.005
Within-subject (per-participant CV)	SVM (RBF)	Raw band power	45.1% ± 13.2%	—	<10^−8^

## Data Availability

De-identified EEG features and analysis scripts are available from the corresponding author upon reasonable request.
